# The Aryl Hydrocarbon Receptor Mediates Leflunomide-Induced Growth Inhibition of Melanoma Cells

**DOI:** 10.1371/journal.pone.0040926

**Published:** 2012-07-17

**Authors:** Edmond F. O’Donnell, Prasad Rao Kopparapu, Daniel C. Koch, Hyo Sang Jang, Jessica Lynne Phillips, Robert L. Tanguay, Nancy I. Kerkvliet, Siva Kumar Kolluri

**Affiliations:** 1 Cancer Research Laboratory, Oregon State University, Corvallis, Oregon, United States of America; 2 Department of Environmental and Molecular Toxicology, Oregon State University, Corvallis, Oregon, United States of America; 3 Molecular and Cellular Biology Program, Oregon State University, Corvallis, Oregon, United States of America; 4 Environmental Health Sciences Center, Oregon State University, Corvallis, Oregon, United States of America; 5 Oregon State University, Corvallis, Oregon, United States of America; Duke University Medical Center, United States of America

## Abstract

A novel role of the dihydroorotatedehydrogenase (DHODH) inhibitor leflunomide as a potential anti-melanoma therapy was recently reported *(Nature 471∶518-22, 2011).* We previously reported that leflunomide strongly activates the transcriptional activity of the Aryl Hydrocarbon Receptor (AhR). We therefore tested whether the AhR regulates the anti-proliferative effects of leflunomide in melanoma. We first evaluated the expression of AhR in melanoma cells and found that AhR is highly expressed in A375 melanoma as well as in several other cancer cell types. To evaluate whether AhR plays a role in regulating the growth inhibitory effects of leflunomide in A375 cells, we generated a stable cell line from parental A375 cells expressing a doxycycline (DOX) inducible AhR shRNA. Using these cells in the absence or presence of DOX (normal AhR levels or AhR-knockdown, respectively) we found that the anti-proliferative effects of leflunomide, but not its metabolite A771726, were strongly dependent upon AhR expression. It has been well established that supplementation of cells with exogenous uridine completely rescues the anti-proliferative effects due to DHODH inhibition. Thus, we performed uridine rescue experiments in A375 cells to determine whether the anti-proliferative effects of leflunomide are solely due to DHODH inhibition as previously reported. Interestingly, saturating levels of uridine only modestly rescued A375 cells from the anti-proliferative effects of both leflunomide and A771726, indicating additional mechanism(s), apart from DHODH inhibition are responsible for the anti-proliferative effects of leflunomide in melanoma cells. Uridine also did not rescue MDA-MB-435S melanoma cell proliferation after leflunomide treatment. Our results reveal that the AhR is a molecular target of leflunomide and support the feasibility of the clinical application of leflunomide for treating melanoma. Furthermore, analysis of expression data from 967 cancer cell lines revealed that AhR is expressed in multiple different cancer types supporting the intriguing possibility of targeting the AhR for therapy in a number of cancers.

## Introduction

The Aryl Hydrocarbon Receptor (AhR) is a ligand activated transcription factor belonging to the basic helix-loop-helix PER/ARNT/SIM (bHLH/PAS) family of transcription factors and regulates a wide range of biological activities ranging from transcriptional modulation of a battery of genes involved in xenobiotic metabolism, most notably members of the cytochrome P450 family, to cell cycle progression through both ligand dependent and independent mechanisms. [1]–[7] The AhR is localized in the cytosol, and upon activation by a ligand translocates to the nucleus where it binds its obligate heterodimeric partner AhR Nuclear Translocator protein (ARNT). This complex proceeds to bind AhR/xenobiotic response elements to regulate the transcription of a battery of target genes in a ligand dependent manner.

A novel and clinically important role for the AhR as a target for anti-cancer therapies has emerged from its classically studied role as a mediator of the effects of environmental toxins such as 2,3,7,8-tetrachlorodibenzo-p-dioxin (TCDD). Recently, the AhR has been shown to act as a tumor suppressor in a mouse model of prostate cancer. [8], [9] Selective AhR modulators that interfere with estrogen receptor transcription have been shown to inhibit breast cancer cell proliferation. [10]–[12] The AhR has also been shown to suppress diethylnitrosamine (DEN) induced liver cancers in the absence of exogenous ligands, [13] and the Aryl Hydrocarbon Receptor Repressor, itself an AhR-target gene, has been shown to mediate tumor suppression in tissues derived from multiple human cancers including those of the colon, breast, lung, stomach, cervix, and ovaries. [14] At the cellular level, the AhR can inhibit proliferation through several distinct mechanisms. [5], [15] In a ligand and cell-type specific manner, activation of the AhR increases expression of CDK inhibitors p27^Kip1^ and p21^Cip1^. [2], [16] The AhR has also been shown to interact with retinoblastoma protein to induce cell cycle arrest by enhancing repression of E2F-dependent transcription. [4]

A few FDA approved drugs have recently been shown to activate AhR transcription. [17]–[19] For example, we recently reported that AhR activation by leflunomide, a well known immunosuppressive agent used to treat rheumatoid arthritis, alters cell proliferation and tissue regeneration in a context-specific manner. [18], [20] Leflunomide is converted to its primary metabolite A771726 via isoxazole ring cleavage, and whereas metabolism of leflunomide to A771726 is required for dihydroorotatedehydrogenase (DHODH) inhibition, [21] this conversion significantly abrogates the AhR-activating properties of leflunomide. [18]

Recently, White et al. reported that DHODH modulates transcriptional elongation in melanoma, and that inhibition of DHODH by leflunomide may be an effective anti-melanoma therapy. [22] Development of new therapeutic approaches for the treatment of melanoma is important, as melanoma accounts for approximately 75% of all skin-cancer related deaths. [23] Interestingly, expression of AhR has been observed in both primary human melanocytes as well as FM55 melanoma cells, in which the endogenously produced tryptophan metabolite 6-formylindolo[3,2-b]carbazole (FICZ) has been demonstrated as a putative AhR ligand capable of regulating melanogenesis in an AhR-dependent manner. [24], [25] The study by White et al. prompted us to test whether the AhR has a role in regulating the effects of leflunomide in melanoma. [22] Our results revealed that the AhR is essential in mediating the anti-proliferative effects of leflunomide in melanoma cells and that the inhibition of DHODH by leflunomide’s active metabolite A771726 can only partially account for inhibition of melanoma cells. Analysis of expression data from 967 cancer cells revealed that AhR is broadly expressed in several cancer types including lung, breast, liver, stomach and pancreas. [26]

## Materials and Methods

### Cell Culture

Maintenance of HEK293T cells and WT Hepa1c1c7 cells (herein Hepa1) in our laboratory has been described previously. [18], [27], [28] HEK293T, MDA-MB-435S, Hepa1, and A375 cells were purchased from American Type Culture Collection (ATCC, Manassas, VA). Cells were cultured in Dulbecco’s Modified Eagle Medium with L-glutamine (Mediatech Inc., Manassas, VA) supplemented with 10% FBS (Tissue Culture Biologicals, Tulare, CA), 100 U/mL penicillin, and 100 mg/mL streptomycin (Mediatech Inc., Manassas, VA) at 37°C with in a humidified 5% CO_2_ atmosphere. Cells were routinely passaged at a dilution of 1∶5 every 2–3 days.

### Chemicals & Reagents

Preparation of leflunomide and A771726 was as previously described. [18] Briefly, both compounds were dissolved in DMSO to a final concentration of 100 mM. All other chemicals and reagents were purchased from Sigma (St Louis, MO) unless otherwise indicated. The final concentration of DMSO was 0.1% v/v in all cell culture assays.

### Inducible AhR Knockdown in A375 Cells

293T cells in 15 cm dishes at ∼60% confluence were co-transfected by calcium phosphate precipitation with 20 µg of a pTRIPZ vector (Openbiosystems, Thermo Scientific) expressing an shRNA against AhR (sense, 5′-CGGGCTCTTTCAAGATAGTAAA′3′), 20 µg of the packaging vector psPAX2, and 10 µg of the envelope vector pMD2.G (Addgene). Media was replaced 24 hours after transfection, and supernatants containing lentivirus particles were collected after a further 24 hours and applied to A375 cells with 8 µg/mL polybrene (Sigma). 24 hours later, cell medium was exchanged with fresh medium containing 3 µg/mL of puromycin to select transduced cells. Non-infected cells were included as a control for puromycin toxicity, and only after 100% of the cells had perished was the selection process considered complete. In addition, stable cell lines were maintained in puromycin until sufficient freezer stocks were generated. Cells were maintained in puromycin for at least one week after thawing. BSL-2 protocols were used for the handling of all virus particles and infections. The final generated cells, A375-pTRIPZ-shAhR, were cultured as described above for A375 cells, except that for AhR knockdown experiments, media containing 2 µg/mL doxycycline (DOX) was exchanged every other day. Non-induced (i.e. no-DOX) cells were maintained as controls. Expression of RFP throughout knockdown experiments was routinely confirmed by either fluorescence microscopy or flow cytometry, while AhR knockdown status at the beginning and end of experiments was routinely verified by Western blot.

### Western Blotting

Analysis of protein abundance was performed by Western blot according to standard techniques. Briefly, cells were collected by trypsinization, resuspended in 1 volume of PBS, lysed in an equal volume of 2X Laemmli Sample Buffer, boiled for 5 min, and stored at −80°C until needed. Lysates were subjected to SDS-PAGE on 4–12% pre-cast XT-Criterion gradient gels (Biorad, Hercules, CA) and transferred to PVDF membranes by semi-dry transfer. Afterwards, blots were blocked in 5% TBSTM and incubated overnight at 4°C with primary antibodies. Primary antibodies were as follows: rabbit-anti-AhR was from Enzo Life Sciences (Farmingdale, NY) and mouse-anti-GAPDH was from SCBT (Santa Cruz, CA); all other antibodies (mouse anti-CDK6, mouse anti-CDK4, mouse-anti-p21, mouse-anti-p27, mouse-anti-Cyclin-D1, and mouse-anti-Cyclin-D3) were purchased from Cell Signaling Technology (Danvers, MA). After incubation with primary antibodies, blots were washed with 0.1% TBST three times for 5 minutes each, followed by incubation at room temperature with goat-anti-mouse or goat-anti-rabbit antibodies (Southern Biotech, Birmingham, AL) conjugated with HRP in 5% TBSTM. Washing was repeated as above, and blots were visualized with SuperSignal West Pico chemiluminescent agent (Pierce Biotechnology/Thermo Scientific, Rockford, IL) using a ChemiGenius Bio Imaging System and Gene Snap Software (Synoptics LTD, Cambridge, UK).

### Gene Expression Analysis

Collection of total RNA and reverse transcription reactions were performed exactly as described previously. [18], [27] Quantitative Polymerase Chain Reaction (qPCR) analysis was performed using an ABI7500 Fast Real-Time PCR system (ABI, Foster city, CA) with RT^2^ Real-Time™ SYBR Green/Rox PCR master mix (SA Biosciences/Qiagen, Frederick, MD) according to the manufactures recommended protocol. Briefly, 25 µL reactions (12.5 µL 2X master mix, 1 µL cDNA, 1 µL 10 mM forward & reverse primer mix, and 10.5 µL nuclease free water) were cycled at 95°C for 10 minutes, followed by 40 cycles consisting of 95°C for 15 seconds and 60°C for 1 minute. Melt-curve analysis was performed at the end of each qPCR experiment to verify single amplicons, and the correct length of PCR products was verified by EtBr gel electrophoresis. The primers used in this study are as follows: *CYP1A1*, Forward, 5′-CTT CAC CCT CAT CAG TAA TGG TC-3′, Reverse 5′-AGG CTG GGT CAG AGG CAA T-3′; *CDKN1A*, Forward, 5′- TGT CCG TCA GAA CCC ATG C -3′, Reverse 5′- AAA GTC GAA GTT CCA TCG CTC -3′; *C-Myc*, Forward, 5′- GGC TCC TGG CAA AAG GTC A-3′, Reverse 5′- AGT TGT GCT GAT GTG TGG AGA-3′; *GAPDH* Forward, 5′-ACC TTT GAC GCT GGG GCT GG-3′, Reverse, 5′-CTC TCT TCC TCT TGT GCT CTT CGT GG -3′. Reactions were performed in triplicate, and data was analyzed by the ΔΔCt method.

### Real-time Cell Analysis

Real time analysis of cellular proliferation was performed using a Real-Time Cell Analyzer (RTCA) DP instrument with E-plate 16 assay platforms (Roche, USA). Measurement and basic analysis of real time analysis data was performed essentially according to the manufactures recommended protocol. Briefly, A375-pTRIPZ-shAhR cells that had never been exposed to doxycycline (AhR proficient) or had been induced for ≥72 hours with 2 µg/mL doxycycline (AhR knockdown) were seeded at a density of 2000 cells/well in a final volume of 200 µL with or without DOX as above. Prior to addition of 100 µL of media containing cells, the instrument was blanked with 100 µL of media. After approximately 18 hours, or when the cells appeared to be entering an exponential growth phase, compounds were added via addition of 20 µL of an 11X stock. DOX was added to the appropriate wells after 48 hours to maintain knockdown. In all experiments, water, the solvent for doxycycline, was added to no-DOX cells as a control.

### Cellular Viability Assay

Cell viability assays were performed as described previously, with modification. [22] Viability was determined using the CellTiter Glo Luminescent Cell Viability Assay (Promega, Madison, WI). Luminescence readings were recorded as previously described. [18], [27] Verification of non-confluent cell status in experiments (i.e. vehicle control wells) was determined by visual assessment (phase contrast microscopy).

### Electrophoretic Mobility Shift Assay (EMSA)

To study the ligand-induced AhR binding to DNA, EMSA was performed as described previously. [29] Briefly, whole cell lysate (16 µg of protein) from human melanoma A375 cells were pre-incubated with A771726 or DMSO for 30 minutes at RT, and incubated with leflunomide or TCDD for 2 hours at RT. The liganded lysates were incubated with ^32^P-labeled double stranded oligonucleotide containing mouse Cyp1a1 xenobiotic responsive element (XRE) for 15 minutes at RT, which was separated by native gel electrophoresis. The dried gel was exposed to the Phospho Imager to visualize the signal.

### Uridine Rescue of DHODH Inhibition

Fresh uridine stock solutions were prepared daily to a concentration of 100 mM in 10% DMEM. Preparations were filter sterilized with a 0.22 µm filter before use in cell culture.

### CFSE Staining

For CFSE analysis, cells were harvested, washed with PBS, and resuspended at a density of 1×10^7^ cells/ml. CFSE was then added to the suspension to a final concentration of 1 uM and incubated for 8 minutes at RT. A 25% volume equivalent of FBS or DMEM with 10%FBS was added to stop the labeling. Cells were washed 2X with PBS and counted for plating. Cells were removed prior to and after staining to verify CFSE incorporation. Post treatment cells were harvested and washed twice in PBS and resuspended in 100 uL BD cytofix and incubated on ice for 1 hour. Cell were then washed twice with PBS and stored at 4°C until analysis. The entire procedure was carried out in dark. Flow cytometry analysis was performed as described previously. [28]

### Cancer Cell Line Encyclopedia

Gene expression data for AhR and DHODH across a panel of 967 cancer cell lines were downloaded from the Cancer Cell Line Encyclopedia (CCLE). [26] The mutational status of BRAF (V600E) in MDA-MB-435S cells was also determined using the CCLE.

### Statistical Analysis

Data were analyzed by one-way ANOVA with either Tukey’s or Bonferroni’s post-test using Prism software (Version 5.0, Graphpad, La Jolla, CA). Values of P<0.05 were considered statistically significant.

## Results

### AhR Signaling is Intact in A375 Melanoma Cells

**Figure 1:**
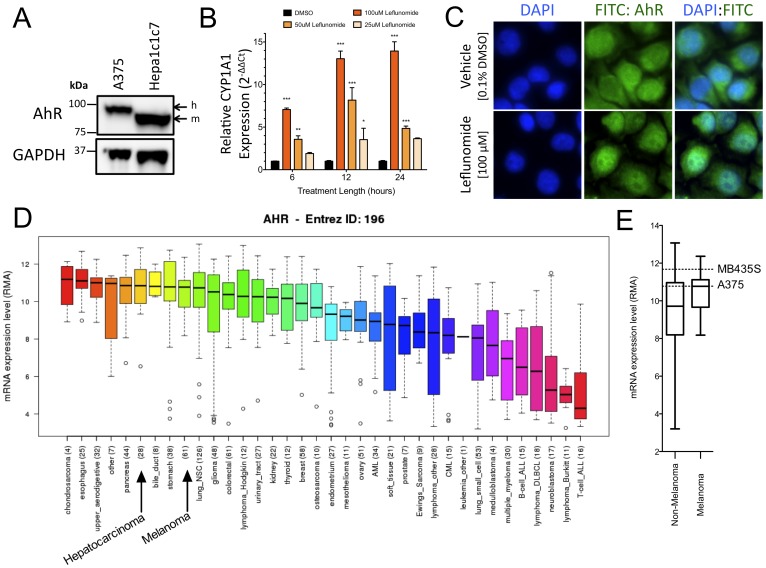
Intact AhR signaling in A375 melanoma cells. (A) Western blot comparing relative abundance of AhR in human A375 melanoma cells and mouse Hepa1c1c7 hepatoma cells; h and m indicate the position of human and mouse AhR isoforms, respectively. GAPDH is shown as an equal loading control. (B) qPCR analysis of CYP1A1 induction in A375 melanoma cells after 6, 12, and 24 hours of treatment with vehicle (0.1% v/v DMSO), or leflunomide at the indicated concentrations. Data are the mean ± SD of 3 independent experiments *** P<0.0001, ** P<0.001, * P<0.01. (C) Leflunomide-induces nuclear localization of the AhR. A375 cells were treated for 4 hours with DMSO or leflunomide (100 µM) and AhR localization was visualized. (D) AhR mRNA expression profile (Log_2_, RMA) of 967 cancer cell lines. Expression profiles of AhR in melanoma cells are compared with liver cancer cells, which have high AhR expression. (E) Box and whisker plots (error bars: min/max expression) of cancer cell line encyclopedia AhR mRNA data for non-melanoma and melanoma extracted from (D). Expression of AhR in melanoma cells vs all other cancer cell types are indicated. The AhR mRNA abundance of the two cell lines used in the study, MDA-MB-435 and A375, are depicted by dashed lines.

In the present study, we hypothesized that the effects of leflunomide in A375 melanoma cells are mediated by a previously unappreciated activation of the AhR. We first confirmed the expression of AhR in A375 melanoma cells by Western blot. A375 cells expressed a significant level of AhR; Mouse Hepa1 cells were used as positive control for AhR expression (Figure 1A). To determine whether AhR signaling is functional in A375 cells, we next tested whether the AhR ligands could upregulate expression the AhR target gene CYP1A1 in a time and dose-dependent manner (Figure 1B). At 6, 12, and 24 hours, treatment of A375 cells with 100 µM leflunomide resulted in a 7.1±0.2, 8.2±0.9, and 13.9±1.1 fold increase in CYP1A1 expression relative to matched vehicle controls (p<0.0001 for all timepoints, n = 3); A dose dependent effect was also apparent at each timepoint. Treatment with 1 nM TCDD for 24 hours also significantly increased CYP1A1 expression in A375 melanoma cells (data not shown). Activation of the AhR by a ligand leads to its nuclear translocation from the cytosol. Treatment of A375 cells with leflunomide resulted in strong nuclear localization of AhR compared to vehicle treated cells (Figure 1C). In addition, we evaluated the expression of AhR across 967 cancer cell lines using the recently developed cancer cell line encyclopedia. [26] We found that AhR is expressed across a wide spectrum of tumor lineage types including those from breast, liver, lung, prostate, stomach, and colorectal cancers. Skin-derived cancer cells exhibited relatively high levels of AhR expression comparable to that of liver cancer cells (Figure 1D). Among skin-derived cancer cells, A375 melanoma cells and MDA-MB-435S cells, [30] which were used in this study, expressed high levels of AhR (figure 1E). Our observation of AhR expression and downstream signaling in melanoma cells is supported by a previous study performed in human melanocytes. [24] Taken together, these data indicate that AhR is highly expressed in transformed melanoma cells and that AhR signaling is activated following treatment with leflunomide in A375 melanoma cells.

### Leflunomide-mediated Inhibition of A375 Cellular Proliferation is Dependent on AhR Expression

**Figure 2:**
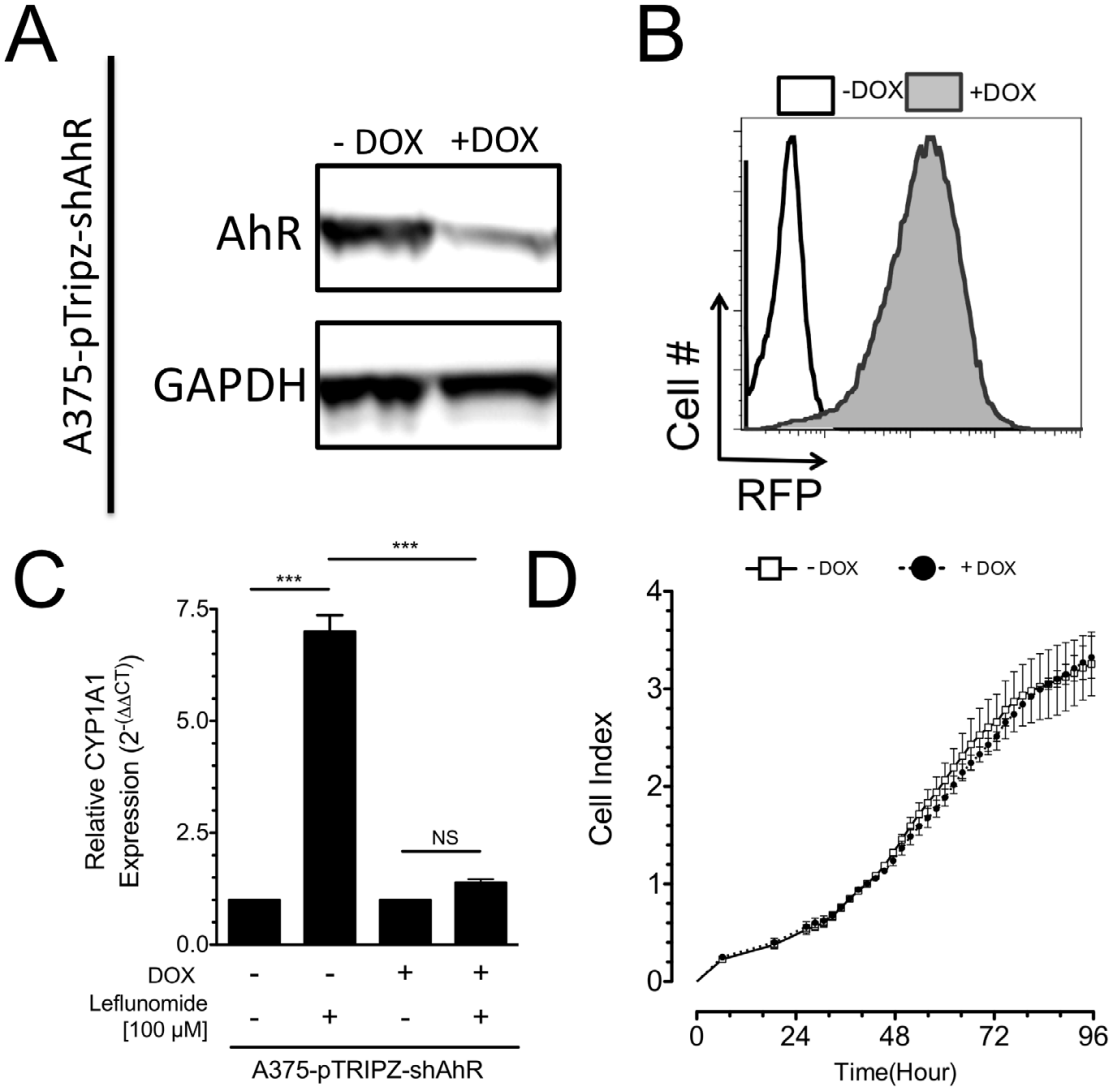
Inducible knockdown of AhR in A375 melanoma cells. (A) Western blot of the A375-pTRIPZ-shAhR cell line in the absence (-) or presence (+) of 2 µg/mL doxycycline (DOX) for 72 hours. GAPDH is shown as an equal loading control. (B) Flow cytometry analysis of red fluorescent protein (RFP) reporter with and without DOX in A375-pTRIPZ-shAhR cells. (C) qPCR analysis of CYP1A1 induction in A375-pTRIPZ-shAhR cells after 24 hours of treatment with vehicle or leflunomide in the absence or presence of DOX. Data are the mean ± SD, *** P<0.0001 NS: Non-significant, n = 3. (D) Real time cellular analysis of A375-pTRIPZ-shAhR proliferation with or without AhR knockdown. Data are the mean ± SD of two biological replicates, and are representative of at least three similar experiments.

We first confirmed that leflunomide inhibits the proliferation of A375 cells as previously demonstrated. [22] At concentrations of 100 and 25 µM, leflunomide strongly decreased viability of A375 cells by approximately 60% and 20% respectively, relative to vehicle (data not shown). We next determined whether the anti-proliferative effects of leflunomide in A375 cells require AhR expression. To this end, we generated an A375 cell line in which AhR expression was controlled by a doxycycline (DOX) inducible shRNA (pTRIPZ-shAhR). Suppression of AhR protein abundance in pTRIPZ-shAhR expressing A375 cells after 72 hours of treatment with 2 µg/mL DOX was confirmed by Western blot (Figure 2A). In addition, a red fluorescent Protein (RFP) reporter also under the regulation of DOX in the same vector was potently induced by DOX as determined by flow cytometry analysis, (Figure 2B) indicating stable, uniform expression of the pTRIPZ-shAhR insert in this cell line. To verify that the AhR knockdown was functionally relevant with respect to target gene activation, we next treated A375 cells expressing pTRIPZ-shAhR cells with leflunomide (100 µM) in the absence or presence of DOX (normal AhR expression and AhR knockdown, respectively) and evaluated the effect of decreased AhR expression on CYP1A1 induction. Consistent with the data in Figure 1B, expression of CYP1A1 was strongly induced by leflunomide in cells expressing AhR, but was significantly attenuated upon AhR knockdown (P<0.001 Leflunomide –DOX vs Leflunomide +DOX, n = 3) (Figures 2A and 2C). A375 cells with normal or reduced expression of AhR did not display apparent differences in morphology, nor did they display noticeably different profiles of growth in culture (Figure 2D).

**Figure 3:**
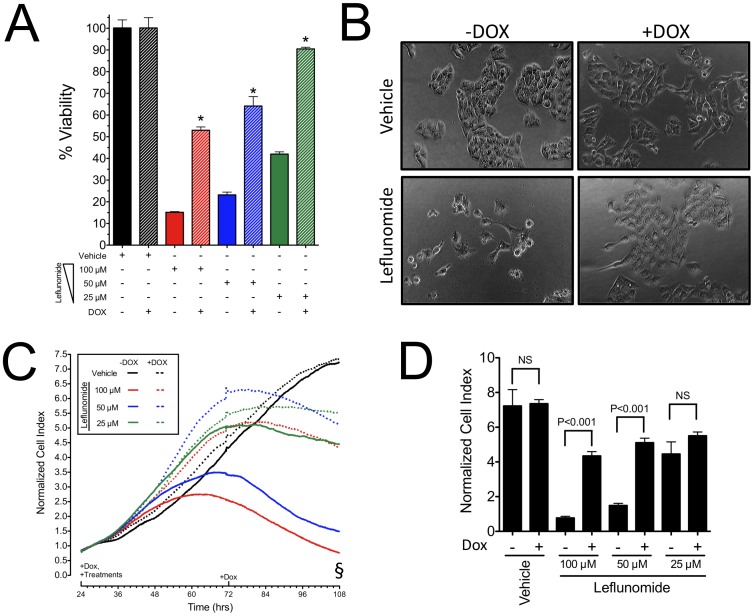
Leflunomide mediated inhibition of A375 melanoma cells is significantly dependent upon AhR expression. (A) Viability of A375-pTRIPZ-shAhR melanoma cells treated with the indicated doses of leflunomide in the absence (AhR-expressing) or presence (AhR-knockdown) of 2 µg/mL doxycycline. Results are the mean ± SD of three independent experiments. N = 6; * P<0.0001 compared to respective dose of leflunomide in the absence of doxycycline. (B) Phase-contrast microscopy images of A375-pTRIPZ-shAhR cells treated with either vehicle or leflunomide (100 µM) for 72 hours in the absence or presence of doxycycline. (C) Real time cellular analysis of A375-pTRIPZ-shAhR proliferation with or without AhR knockdown in the presence of vehicle (0.1% v/v DMSO) or leflunomide at concentrations of 100, 50, or 25 µM. Data are the mean of two biological replicates and are representative of two independent experiments. (D) Cell index values at the end of the real-time analysis period (§) were evaluated by ANOVA.

Having verified the ability to induce knockdown of AhR in A375 cells and demonstrating that suppression of AhR expression abolishes AhR-mediated induction of CYP1A1 by leflunomide, we next evaluated the effect of AhR knockdown on proliferation of A375 cells treated with leflunomide. First, A375-pTRIPZ-shAhR cells were treated with 100, 50, and 25 µM leflunomide with or without DOX, and cell viability was determined after 96 hours (Figure 3A). The percent of viable A375 cells exposed to 100, 50, and 25 µM leflunomide relative to vehicle treated cells were 15.2±0.8%, 23.2±3.2%, and 42.0±2.5%, whereas suppression of AhR expression increased A375 cell viability to 53.0±3.7%, 64.2±10.7%, and 90.5±1.9%, respectively (Mean ± SD, p<0.0001 for all treatments). Phase contrast microscopy also revealed a noticeable difference between A375 cells treated with leflunomide with normal or reduced AhR protein levels (Figure 3B).

To further evaluate the AhR-dependent anti-melanoma effects of leflunomide, we next examined the role of AhR in mediating the anti-proliferative effects of leflunomide using xCELLigence system, which monitors the combined parameters of cellular proliferation and morphology in real-time through an electrical-impedance based measurement calculated as a cell index. [31] A375 cells with normal or reduced AhR expression were treated with leflunomide at concentrations of 100, 50, 25 µM and proliferation was compared relative to a vehicle control. Consistent with the results of our endpoint analysis of A375 cell viability, A375 cells with reduced AhR expression had much higher cell index at all time points compared to A375 cells expressing AhR (Figure 3C). We also compared the cell index of treatments at the end of the assay by ANOVA, and significant differences in cell indices were observed for treatments of 100 and 50 µM leflunomide with or without AhR (Figure 3D, p<0.001).

**Figure 4:**
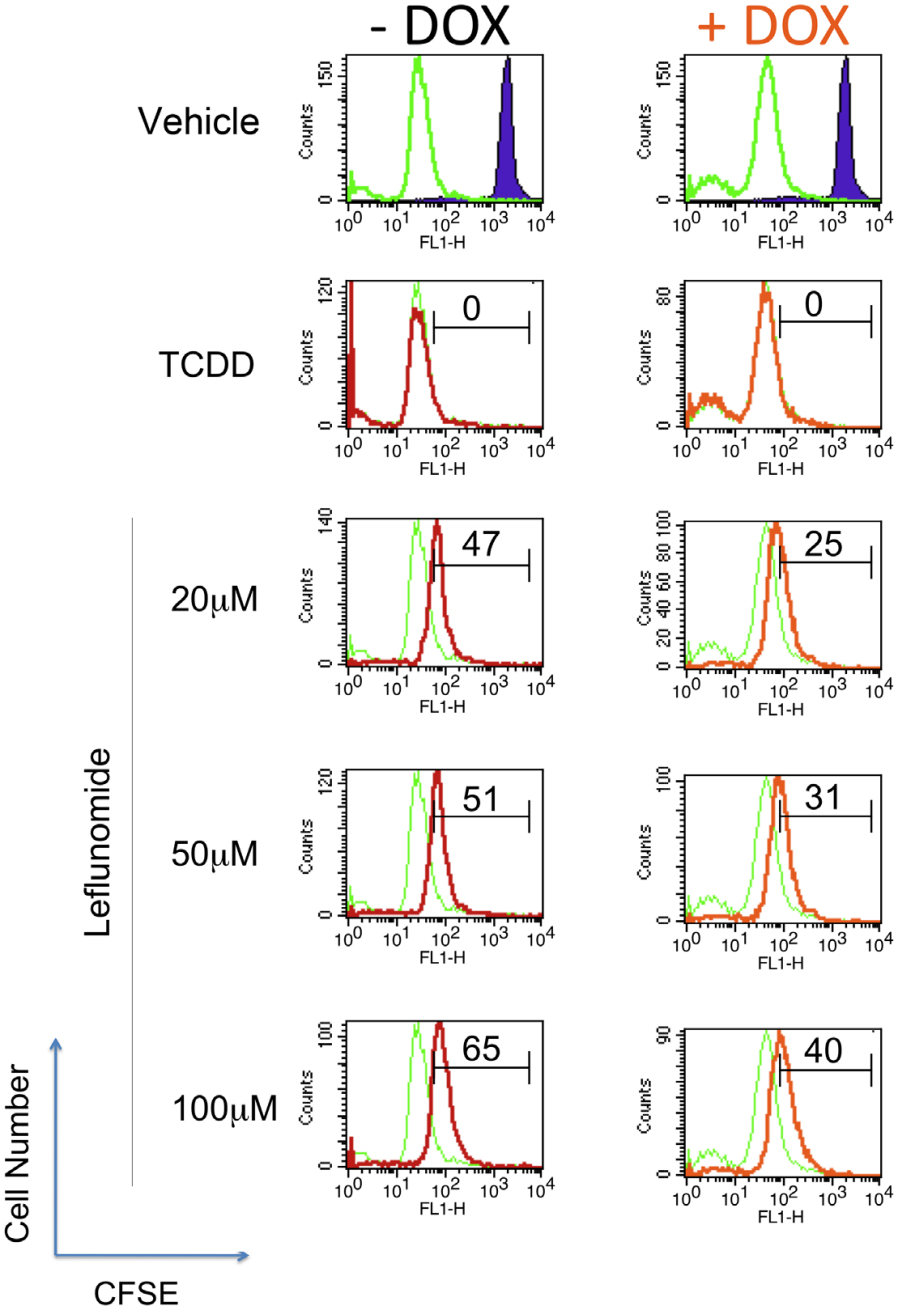
Analysis of CFSE-stained A375 cells treated with leflunomide confirms an AhR-dependent effect on proliferation. A375-pTRIPZ-shAhR cells with or without DOX induced-AhR knockdown were stained with CFSE and treated with vehicle (DMSO 0.1% v/v), 1 nM TCDD, or leflunomide at 20, 50, and 100 µM for 72 hours followed by flow cytometry. Vehicle treated cells (top panels, green histograms) exhibited decreased CFSE fluorescence intensity compared to cells before the start of treatments (‘time 0’) (solid histograms), reflecting dilution of CFSE in vehicle-treated cells and reflecting cell division. Numbers indicate the percent difference between vehicle (green) and treatment (Red -Dox; Orange +Dox) within histogram plots.

In addition to xCelligence monitoring, we also utilized carboxyfluorescein diacetate succinimidyl ester (CFSE) staining to track proliferating A375-pTRIPZ-shAhR cells by flow cytometry. Dilution of CFSE fluorescence in vehicle treated cells was readily apparent at 72 hours compared to cells stained at the start of the treatments (‘day 0’) (Figure 4, top panels). After 72 hours, a dose-dependent increase in CFSE fluorescence intensity, which is indicative of fewer cellular divisions, was observed in leflunomide treated samples. Consistent with our viability and real-time cell monitoring, this effect was significantly decreased in DOX-treated cells, indicating that suppression of AhR expression led to more cellular divisions compared to cells expressing normal levels of AhR, after leflunomide treatment. Specifically, the difference in CFSE fluorescence intensity between vehicle treated cells and cells treated with 100, 50, and 20 µM leflunomide for 72 hours was 65%, 51%, and 47%, respectively. However, suppression of AhR expression strongly shifted CFSE fluorescence intensity towards the vehicle control, such that treatment with 100, 50, and 20 µM leflunomide under the same conditions produced a much smaller difference in CFSE fluorescence relative to vehicle controls (40%, 31%, and 25%, respectively; Figure 4). Interestingly, no difference in CFSE fluorescence was observed when cells were treated with 1 nM TCDD at either time-point, regardless of AhR expression. Taken together, our analysis of the effects of leflunomide in A375 cells revealed that AhR knockdown conferred a significant resistance to the anti-proliferative effects of leflunomide in A375 melanoma cells by three independent assays, strongly indicating that the AhR regulates the anti-proliferative effects of leflunomide in melanoma cells.

### A771726 Inhibition of A375 Melanoma Cells is Independent of AhR

**Figure 5:**
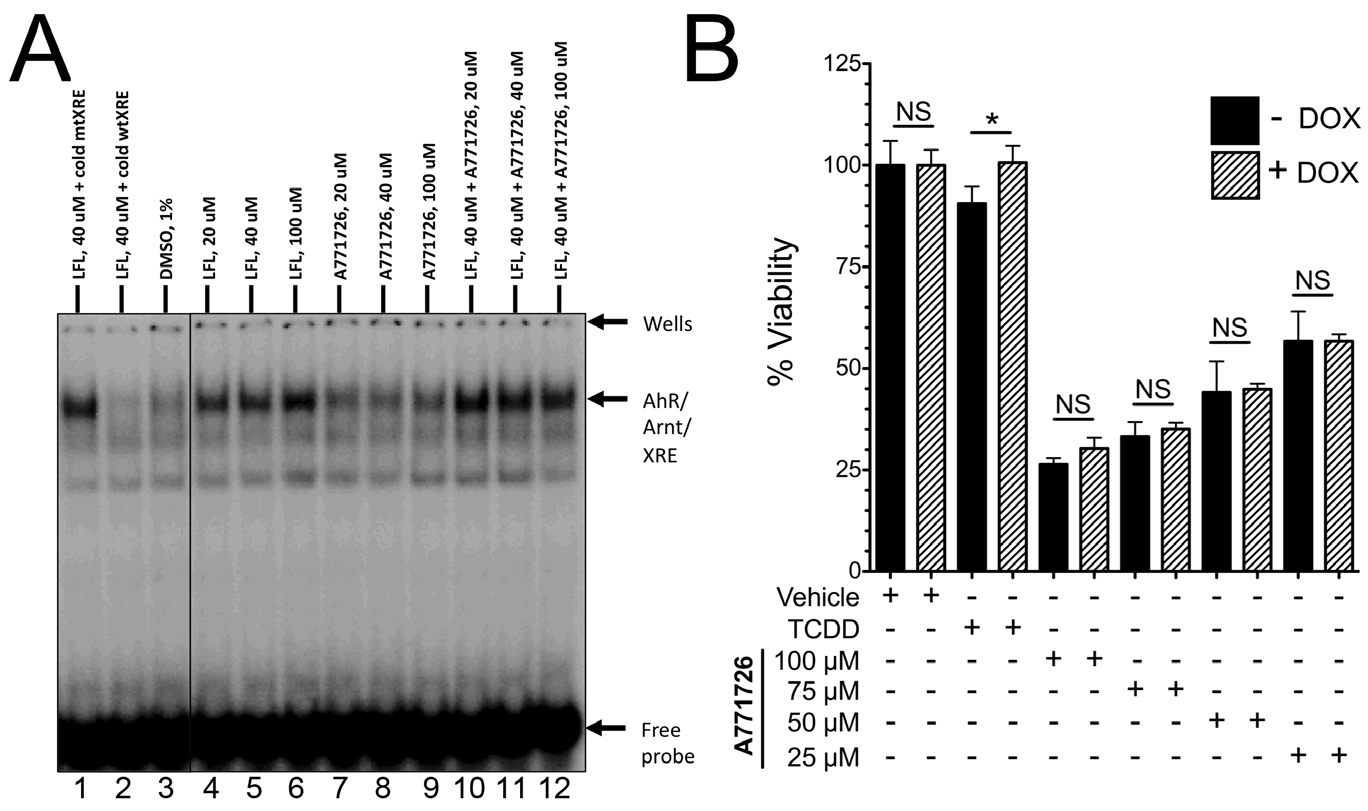
The anti-proliferative effects of A771726 are AhR independent. (A) Leflunomide, but not A771726 induce DNA-binding of the AhR and A771726 does not antagonize leflunomide-induced DNA binding of the AhR. Whole cell extracts (16 µg of protein) from human melanoma A375 cells were pre-incubated with or without A771726 or DMSO for 30 minutes at room temperature (RT), followed by incubation with leflunomide at the indicated concentrations for 2 hours at RT. The resulting lysates were incubated with ^32^P-labeled double stranded oligonucleotide containing mouse CYP1A1 xenobiotic responsive element (XRE) for 15 minutes at RT, which was separated by native gel electrophoresis. The dried gel was exposed to Phosphor Imager to visualize the signal. As a specificity control, 10 ng unlabeled (cold) wild type (wtXRE) or mutant (mtXRE) probes were incubated with the lysates treated with leflunomide for 15 minutes at RT, followed by incubation with ^32^P-labeled probe. (B) Inhibition of A375 melanoma cell proliferation by A771726 is independent of AhR expression. Viability of A375-pTRIPZ-shAhR cells with or without doxycycline was measured 96 hours after treatment with the indicated concentrations of A771726. NS, not significant with respect to the indicated comparison; * P<0.05, Data are the mean ± SD of three independent experiments.

We previously reported that metabolic conversion of leflunomide to A771726 abrogates its ability to activate the AhR in hepa1 hepatoma cells. [18] To confirm and extend this finding to A375 melanoma cells, we performed electrophoretic mobility shift assays (EMSA) using whole cell extracts of A375 cells and XRE sequence containing DNA probes. Incubation of A375 cell extracts with leflunomide resulted in a strong XRE-band compared with DMSO treatment (Figure 5A, lane 3 vs lanes 4–6), whereas A771726 failed to generate such a signal (Figure 5, lanes 4–9). Specificity of signal was confirmed by the addition of non-labeled (cold) wildtype (wt) XRE-probe (Figure 5A, lane 2), which specifically inhibited the leflunomide-induced XRE gel shift. A cold mutant XRE probe had no effect on leflunomide-induced gel shift (Figure 5A, lane 1). In addition, pre-incubation of A375 cell extracts with three different concentrations of A771726 did not antagonize the ability of leflunomide-induced AhR DNA binding. Given that the anti-proliferative effects of leflunomide are dependent upon the AhR, the inability of A77176 to activate the AhR suggests that AhR is not involved in the growth inhibition of A375 cells by A771726. Consistent with this prediction, A375 cells expressing pTRIPZ-shAhR with or without DOX did not exhibit any differences in proliferation upon treatment with various doses of A771726 ranging from 25 to 100 µM (Figure 5B). Thus, while A771726 is also able to inhibit A375 melanoma cells, these effects are independent of the AhR expression.

### The Anti-proliferative Effects of Leflunomide in A375 Melanoma Cells are only Partially DHODH-Dependent

**Figure 6:**
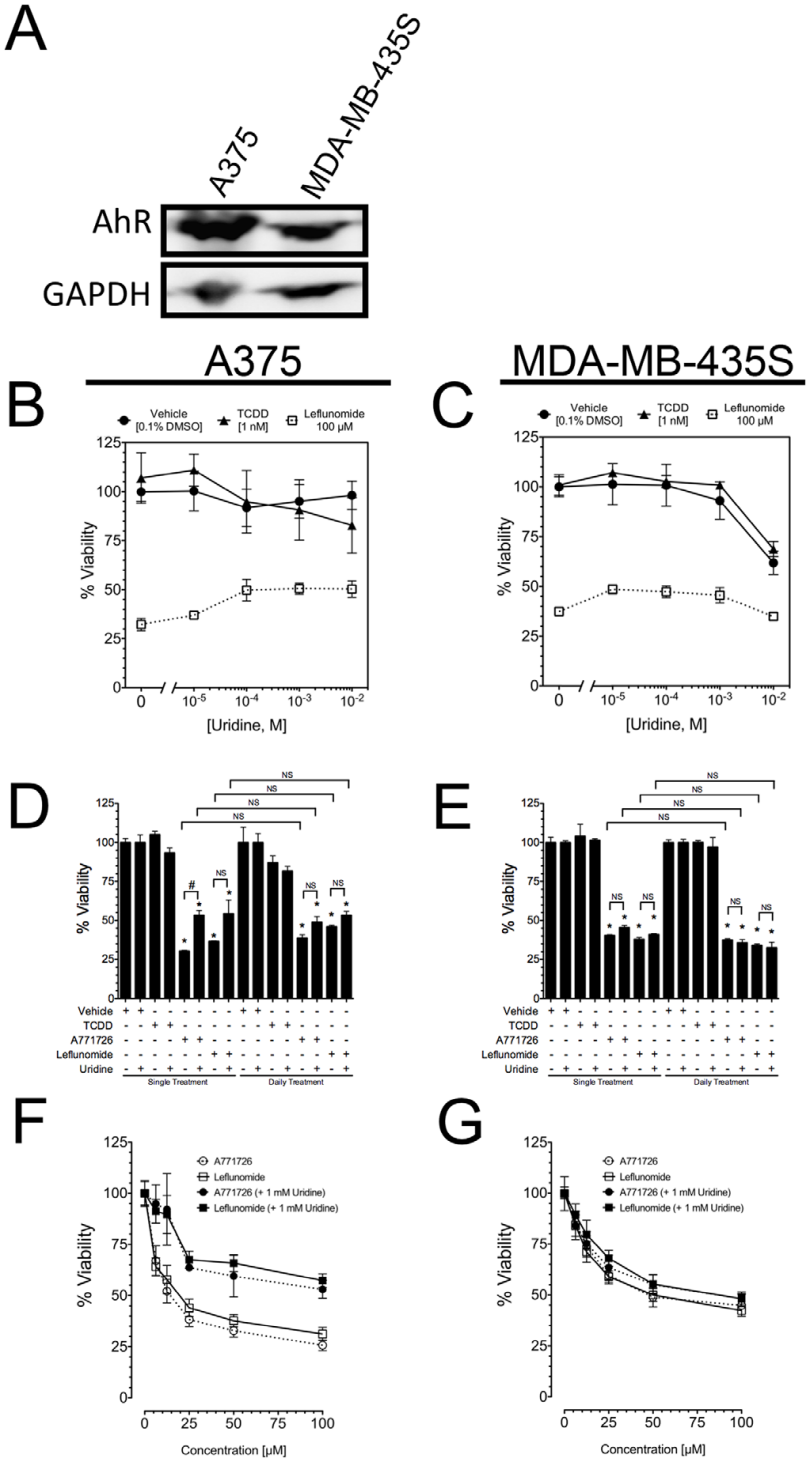
Supplementation with exogenous uridine only partially rescues melanoma cells from the anti-proliferative effects of leflunomide. (A) Relative AhR expression in A375 and MDA-MB-435S melanoma cell lines was analyzed by Western blot; GAPDH was used as a loading control. (B-C) Viability of A375 (B) and MDA-MB-435S (C) cells treated with either vehicle (0.1% v/v DMSO), TCDD (1 nM), or leflunomide (100 µM) supplemented with a range of concentrations of uridine. (D-E) Viability of A375 (D) and MDA-MB-435S (E) cells supplemented with or without exogenous uridine (1 mM) treated with either vehicle (0.1% v/v DMSO), TCDD (1 nM), A771726 (100 µM) and leflunomide (100 µM). Cells were treated either once (single treatment) with the indicated compounds, or exposed to freshly prepared stock solutions every 24 hours (daily treatment) until the end of the experiment. NS, not significant with respect to the indicated comparison; #, P<0.05 with respect to the indicated comparison; *, P<0.0001 with respect to the corresponding vehicle control, relative to the presence or absence or uridine and treatment as indicated. Data are the mean ± SEM of three independent determinations. (F–G) Leflunomide and A771726 dose-dependent effects in A375 (F) and MDA-MB-435S (G) of cells treated with 1 mM uridine or vehicle. Data are the mean ± SD of three independent experiments.

Dihydroorotate Dehydrogenase (DHODH) is an essential enzyme in the *de novo* pyrimidine biosynthesis pathway, and inhibition of DHODH is mediated by the active metabolite of leflunomide, A771726. Through this mechanism of action, leflunomide is currently used in the clinic to inhibit proliferation of T-cells to produce an immunosuppressive effect useful for the treatment of autoimmune diseases such as rheumatoid arthritis. Importantly, the DHODH inhibitory effects of leflunomide (after its conversion to A771726) are completely rescued by supplementation with exogenous uridine, which can be salvaged to uridine monophosphate (UMP) by uridine kinase**.** [20], [32]–[34] Furthermore, inhibition of DHODH was proposed by White et al as a potential anti-melanoma therapy. [22] To investigate the degree to which DHODH inhibition by leflunomide contributes to the anti-proliferative effects in melanoma cells, we performed uridine rescue experiments in A375 and MDA-MB-435S melanoma cells [30], [35], both of which express abundant levels of AhR (Figure 1E and 6A).

We first performed a uridine dose response rescue in A375 and MDA-MB-MB435S cells to identify the minimum effective concentration of uridine that rescues cells from the anti-proliferative effects of leflunomide at 100 µM. Compared with vehicle-treated A375 cells without exogenous uridine (99.9±4.9%), concentrations of uridine ranging from 10 µM to 10 mM added at the beginning of the assay had little to no effect on A375 viability (100.3±10.1% to 98.2±7.2%, respectively) (Figure 6B). Consistent with our previous observations, treatment of A375 cells with 100 µM leflunomide for 96 hours significantly reduced viability; however, while supplementation with uridine did improve viability, the effect was only partial and reached a plateau with uridine concentrations higher than 100 µM. The viability of A375 cells treated with leflunomide and supplemented with uridine at 10 µM, 100 µM, 1 mM, and 10 mM was 37.0±1.7%, 49.8±5.5%, 50.6±2.8%, and 50.3±4.2%, respectively (Figure 6B). Thus, the maximum rescue of viability of cells by uridine supplementation treated with 100 µM leflunomide was approximately 18%, suggesting DHODH inhibition is only partially responsible for the anti-proliferative effects of leflunomide and other mechanism(s) contributing to the effects of leflunomide. This assay was repeated in MDA-MB-435S cells, which largely recapitulated the results from A375 cells, with the notable exception that 10 mM uridine dramatically reduced viability of vehicle and TCDD treated cells (Figure 6C). Based on these results, a concentration of 1 mM uridine was chosen for use in additional uridine rescue experiments.

To confirm that the partial rescue of cell viability was not due to degradation of uridine during the assay, an additional experiment was performed whereby viability of cells treated with leflunomide or A771726 at 100 µM for 96 hours was evaluated after a single addition of uridine at the beginning of the assay, or by daily replacement of media with freshly prepared uridine and compounds. Consistent with our observation above, cell viability after treatment with leflunomide or A771726 again was rescued to a similar, albeit incomplete, degree following either single or daily supplementation with uridine in A375 and MDA-MB-435S cells (Figures 6D and 6E, respectively). Interestingly, while A375 cells did respond to uridine in terms of a partial increase in viability, no significant increase in viability was observed in MDA-MB-435S cells. We next examined a range of leflunomide and A771726 concentrations (100 µM to 6.25 µM) in the absence or presence of 1 mM uridine. Consistent with the above findings, uridine only partially rescued A375 viability even at lower doses of leflunomide and A771726 (Figure 6F). Likewise, MDA-MB-435S cells were largely unresponsive to uridine supplementation (Figure 6G), strongly supporting a DHODH-independent anti-melanoma activity of leflunomide and A771726.

### Expression of P21^cip1^ is Increased by Leflunomide in A375 Melanoma Cells

**Figure 7:**
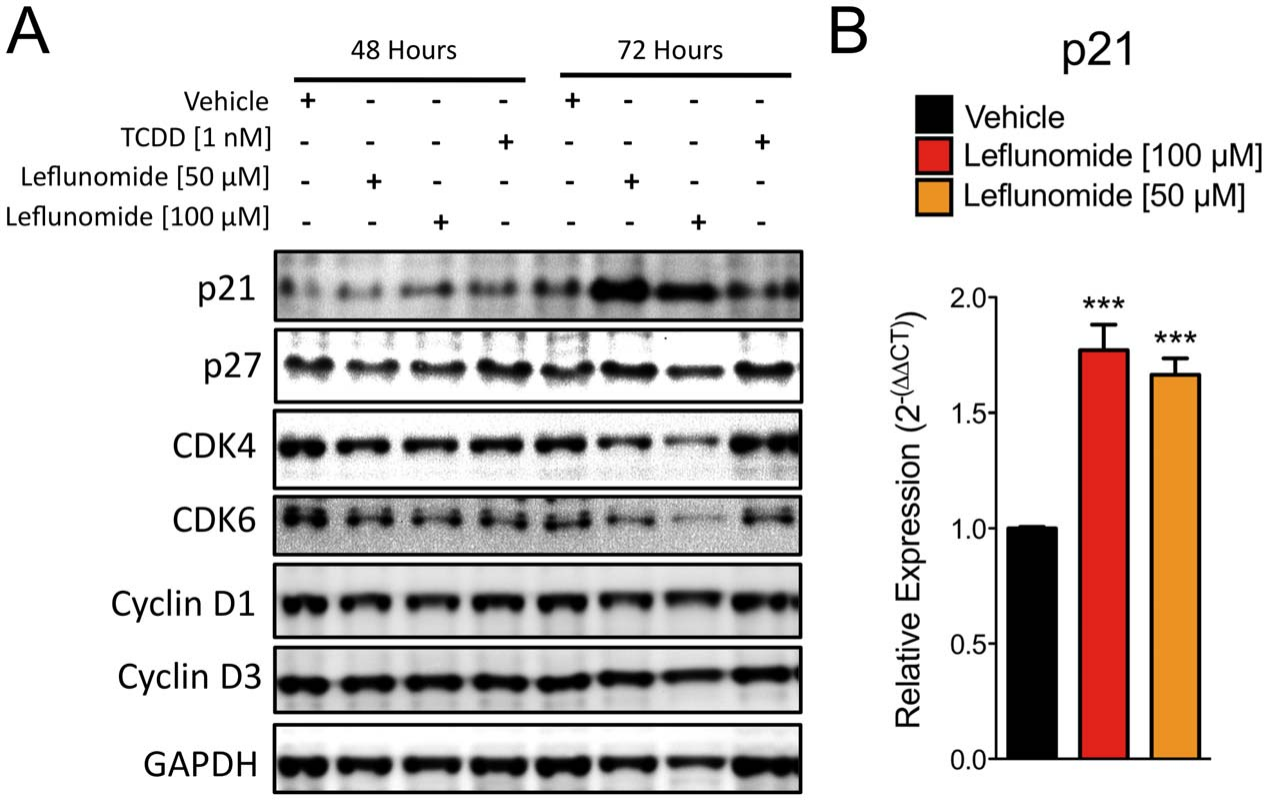
Leflunomide induces expression of p21^Cip1^ in A375 melanoma cells. (A) Protein lysates from A375 cells treated with Vehicle, TCDD, or leflunomide at 100 and 50 µM for 48 and 72 hours were analyzed by Western blot for a panel of cell-cycle regulatory proteins comprising p21, p27, CDK4, CDK6, Cyclin D1, and Cyclin D3. GAPDH was used as a loading control. (B) Expression of p21 mRNA in A375 cells treated with vehicle or leflunomide at 100 and 50 µM was analyzed by qPCR. Data are the mean ± SD, n = 3, # P<0.05, *** P<0.0001.

Having found that uridine only partially rescues melanoma cells from the anti-proliferative effects of leflunomide, we next evaluated potential downstream targets of leflunomide. To this end, A375 cells were treated with either vehicle, 1 nM TCDD, and leflunomide at 50 µM and 100 µM for 48 and 72 hours, after which protein lysates were then collected and analyzed by Western blot to evaluate the effect of leflunomide on expression of several proteins that are known to regulate cell cycle progression, namely, p21^cip1^, p27^kip1^, CDK4, CDK6, Cyclin D1, and Cyclin D3 (Figure 7A). While the expression profiles of the majority of these proteins from this panel were consistent with the GAPDH loading control, a significant increase in p21 expression by leflunomide at 50 and 100 µM was observed (Figure 7A). We also confirmed increase in p21 mRNA expression by leflunomide using qPCR (Figure 7B). Interestingly, p21 has been described previously as a downstream target gene of AhR activation by 3-methylcholanthrene, an AhR ligand, [16] but not TCDD. [36]

### Expression of c-Myc is not altered by Leflunomide in A375 Melanoma Cells

**Figure 8:**
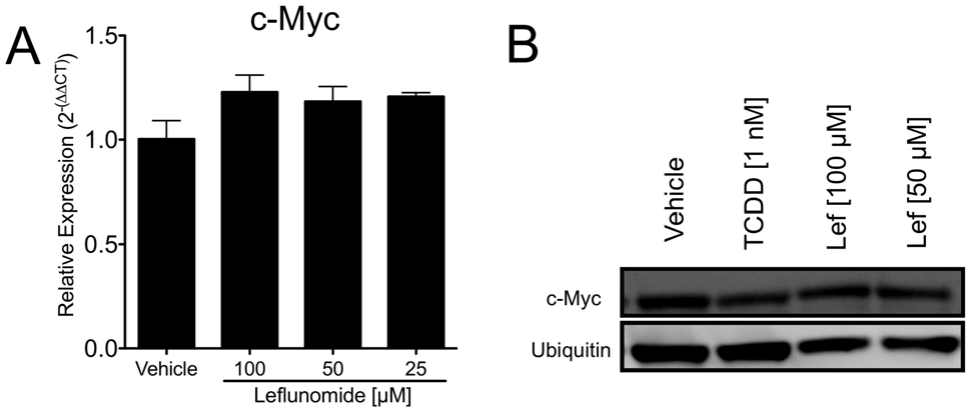
Leflunomide does not alter the expression of c-Myc. (A) qPCR of A375 cells treated for 24 hours with 100, 50, or 25 µM of leflunomide or vehicle (0.1% DMSO). (B) Western blot of A375 cells treated for 48 hours with 100 or 50 µM of leflunomide or 1 nM TCDD.

Lastly, we evaluated the effect of leflunomide on c-Myc expression, which was described as a molecular target of DHODH inhibition by leflunomide due to decreased transcriptional elongation. [22] c-Myc has also been shown to be regulated by the AhR. [37] We did not observe any appreciable changes in c-Myc expression by leflunomide at either the mRNA or protein level (Figures 8A-B).

## Discussion

In the present study, we identified an AhR-dependent inhibition of proliferation of A375 melanoma cells by the clinically-used drug leflunomide (Figure 3), which has been reported previously to activate the AhR. [17], [18], [38] Our results confirm the clinical potential of leflunomide in treating melanoma, and offer insight into its mechanism of action. Inhibition of DHODH was proposed as the mechanism of action for the anti-proliferative effects of leflunomide in melanoma. [22] Knockdown of DHODH expression was shown to inhibit cellular proliferation, which provides only corollary evidence for the effects of leflunomide in melanoma cells, and thus does not provide direct evidence that leflunomide functions by a DHODH-dependent mechanism. [22] To directly test whether DHODH is involved in the anti-proliferative effects of leflunomide, we performed uridine rescue experiments (Figure 6). Our results demonstrated that uridine supplementation only partially rescued A375 cells from the anti-proliferative effects of not only leflunomide, but also A771726 (Figures 4,5,6), strongly suggesting additional mechanism(s) of action of leflunomide in melanoma. In support of this observation, the MDA-MB-435S cell line, which shares the same BRAF^V600E^ mutation as A375 cells, failed to exhibit any significant rescue by uridine with respect to the anti-proliferative effects of leflunomide (Figure 6).

Our observation of a DHODH-independent mechanism of leflunomide is consistent with the different ranges of leflunomide concentrations necessary to achieve inhibition of A375 cellular proliferation and those needed for DHODH inhibition. [20] Likewise, complete rescue of the anti-proliferative effects of A771726 have been demonstrated by uridine previously in non-lymphoid cells including osteosarcoma, rat liver, and smooth muscle cells *in vitro*. [33], [39] Thus, our observation of incomplete rescue of melanoma cell proliferation by uridine strongly suggests additional mechanisms of action of leflunomide and A771726. Additional molecular targets for A771726 have been proposed, including receptor tyrosine kinase inhibition, which may explain why leflunomide, but not A771726, exhibited AhR-dependent anti-proliferative effects.

With respect to the anti-proliferative potential of leflunomide, our findings are largely consistent with those of White et al., confirming its anti-melanoma activity while providing further insight into its mechanism of action. Our results demonstrate that AhR is the molecular target of leflunomide and it is required for leflunomide-induced growth inhibition (Figures 3 and 4). Whether AhR activates a distinct pathway to mediate leflunomide’s anti-proliferative effects or controls conversion of leflunomide into its active metabolite remains to be established. Leflunomide is thought to be rapidly converted to A771726 in the gut and plasma; however, the extent to which first pass metabolism also plays a role in this process is not fully understood. We previously reported that A771726 failed to activate AhR-mediated gene transcription or promote nuclear translocation of the AhR. [18] Consistent with this observation, A771726 did not antagonize leflunomide-induced AhR DNA binding (Figure 5). Thus, our data strongly suggest that the effects of A771726 in melanoma are functionally independent of the AhR. We previously reported that the ring-opening event involved in the conversion of leflunomide to A771726 generates unfavorable docking conditions for A771726 to bind to the AhR. [16] With respect to potential molecular targets of leflunomide in melanoma, we did not observe any changes of c-MYC mRNA or protein expression by leflunomide (Figure 8); however, we did observe significantly increased levels of p21^Cip1^ by leflunomide (Figure 7).

Taken together, our results support the feasibility of using leflunomide to treat melanoma. For the treatment of rheumatoid arthritis, leflunomide is currently administered orally with a loading dose of 100 mg tablets followed by daily ingestion of 10 or 20 mg tablets. A771726 is detected at significant concentrations following oral administration, whereas detection of leflunomide in plasma is significantly lower (Arava® product insert). White et al. were able to successfully use intraperitoneal injections of leflunomide (7.5 mg/kg) to inhibit the growth of A375 xenografts in nude mice (Supplemental Figure 16 of reference [22]). Clinical administration of leflunomide to treat melanoma in humans will likely require alternative routes of administration other than the oral route to achieve effective plasma concentrations of leflunomide. Nevertheless, the recent study by White et al. allowed us to identify an overlooked AhR-dependent anti-cancer effect of leflunomide, and our results support the feasibility of the clinical application of leflunomide in melanoma and provide insight into leflunomide’s anti-proliferative effects.

Lastly, we evaluated AhR expression among 967 cancer cell lines using the newly developed Cancer Cell Line Encyclopedia. [26] As AhR is highly expressed in several cancer types (Figure 1D), tumor suppressive actions of the AhR may be exploited to develop AhR-based therapeutics for several cancers including breast, liver, lung, prostate, stomach, and colorectal cancers.
